# System Architecture for "Support Through Mobile Messaging and Digital Health Technology for Diabetes" (SuMMiT-D): Design and Performance in Pilot and Randomized Controlled Feasibility Studies

**DOI:** 10.2196/18460

**Published:** 2021-03-26

**Authors:** Yuan Chi, Carmelo Velardo, Julie Allen, Stephanie Robinson, Evgenia Riga, David Judge, Lionel Tarassenko, Andrew J Farmer

**Affiliations:** 1 Institute of Biomedical Engineering Department of Engineering Science University of Oxford Oxford United Kingdom; 2 Nuffield Department of Primary Care Health Sciences University of Oxford Oxford United Kingdom

**Keywords:** type 2 diabetes, short message service, health-related behavior, mobile health, mHealth, mobile phone

## Abstract

**Background:**

Diabetes is a highly prevalent long-term condition with high morbidity and mortality rates. People with diabetes commonly worry about their diabetes medicines and do not always take them regularly as prescribed. This can lead to poor diabetes control. The Support Through Mobile Messaging and Digital Health Technology for Diabetes (SuMMiT-D) study aims to deliver brief messages as tailored interventions to support people with type 2 diabetes in better use of their diabetes medicines and to improve treatment adherence and health outcomes.

**Objective:**

This paper describes the overall architecture of a tailored intervention delivery system used in the pilot and randomized controlled feasibility studies of SuMMiT-D and reports its performance.

**Methods:**

The SuMMiT-D system includes several platforms and resources to recruit participants and deliver messages as tailored interventions. Its core component is called the *clinical system* and is responsible for interacting with the participants by receiving and sending SMS text messages from and to them. The personalization and tailoring of brief messages for each participant is based on a list of built-in commands that they can use.

**Results:**

For the pilot study, a total of 48 participants were recruited; they had a median age of 64 years (first quartile, third quartile [*Q*_1_, *Q*_3_: 54.5, 69]). For the feasibility study, a total of 209 participants were recruited and randomly assigned to either the control or intervention group; they had a median age of 65 years (*Q*_1_, *Q*_3_: 56, 71), with 41.1% (86/209) being female. The participants used the SuMMiT-D system for up to 6 months (26 weeks) and had a wide range of different interactions with the SuMMiT-D system while tailored interventions were being delivered. For both studies, we had low withdrawal rates: only 4.2% and 5.3% for the pilot and feasibility studies, respectively.

**Conclusions:**

A system was developed to successfully deliver brief messages as tailored health interventions to more than 250 people with type 2 diabetes via SMS text messages. On the basis of the low withdrawal rates and positive feedback received, it can be inferred that the SuMMiT-D system is robust, user-friendly, useful, and positive for most participants. From the two studies, we found that online recruitment was more efficient than recruitment via postal mail; a regular SMS text reminder (eg, every 4 weeks) can potentially increase the participants’ interactions with the system.

**Trial Registration:**

ISRCTN Registry ISRCTN13404264; http://www.isrctn.com/ISRCTN13404264

## Introduction

Diabetes is one of the most prevalent long-term conditions affecting the world population [1]. Currently, 4.7 million people in the United Kingdom have diabetes; more than 5 million people will have diabetes by 2025. Among people with diabetes, approximately 90% have type 2 diabetes [2]. People with type 2 diabetes are at high risk of developing serious complications, including cardiovascular disease, stroke, and chronic kidney disease, which in turn lead to an increase in the cost and resources needed for health care [1,3,4]. To avoid related comorbidities and complications, treatments with proven efficacy are needed alongside facilitating patients in the self-management of their condition [5-7].

People with diabetes commonly worry about their medicines and do not always take them regularly as prescribed [8]. This can lead to poor diabetes control. To support people with type 2 diabetes in better use of medicines, a diverse range of care programs are available. Many such services are standardized according to available evidence and based on guidelines; however, not all people equally benefit from such a *one-size-fits-all* approach [9-11]. People with poorly controlled diabetes benefit mostly from intensive, provider-driven management, whereas people with adequate glucose levels might maintain these levels independently. A more patient-centered approach is becoming a preferred strategy for improving patient outcomes, where the care is tailored according to individual patient needs and preferences [12-15].

Delivering brief messages that can address a wide range of concerns at a wide scale via digital health systems, in addition to usual care, is a promising approach to develop tailored interventions to improve treatment adherence and health outcomes [16-19]. It has been shown to be both effective and of low cost in improving health in other health conditions, including in reducing cardiovascular risk, lowering blood pressure, and stopping smoking [20-22]. In addition, as tailored interventions are more personally relevant for recipients, they would have a higher chance of being noticed, read, understood, and acted on [23].

This paper describes a SMS-based system developed for and evaluated in the Support Through Mobile Messaging and Digital Health Technology for Diabetes (SuMMiT-D) study. Through mobile health technology integrated with clinical care, this system delivers automated, tailored brief messages to support people with type 2 diabetes in effectively using medicines. The system has currently been used by more than 250 patients with type 2 diabetes across the United Kingdom and is planned to be used by another 1000 patients in the near future. The system is designed to be user-friendly: it is based on carefully developed SMS messages aimed at a large target population and accommodates users without previous experience of digital technologies. The SuMMiT-D system incorporates algorithms that identify messages to be tailored according to the user’s response and characteristics (eg, medical history). This paper addresses an existing gap in the literature: the design of systems to deliver brief messages in the health care delivery system. The messages are tailored and personalized interventions to support people with type 2 diabetes. This paper presents the technical methodology used in the pilot and feasibility studies and reports on the system performance. A paper reporting the protocol for the SuMMiT-D feasibility study [24] has been published separately.

## Methods

### SuMMiT-D

SuMMiT-D is a program of work intended to develop and test the delivery of tailored and personalized brief messages, implemented in SMS text messages. Although implemented in a study, it is a demonstration of how this could be achieved when integrated within the National Health Service (NHS). The aim of the SuMMiT-D system is to provide enhanced and optimal support for people with type 2 diabetes. The program of work reported here consisted of two stages: a formative work package based around a pilot study to develop and test the system and a randomized controlled trial to evaluate feasibility. For the pilot study, 48 participants were recruited to evaluate and assess the system. During this phase, participants were asked to use the SuMMiT-D system for 13 weeks, with the possibility of continuing their participation for up to 26 weeks. This stage aimed to understand the feasibility of the technical aspects of the study.

For the second phase, a feasibility study, 209 participants were recruited. This study evaluated the use of the system for a total of 6 months (26 weeks). Its purpose was to understand the participant recruitment rate and timing as well as the feasibility of integrating the developed system directly with general practitioner (GP) systems. This work was completed in October 2019. The developed methodology will be used for the main trail in 2020.

### The SuMMiT-D System

**Figure 1:**
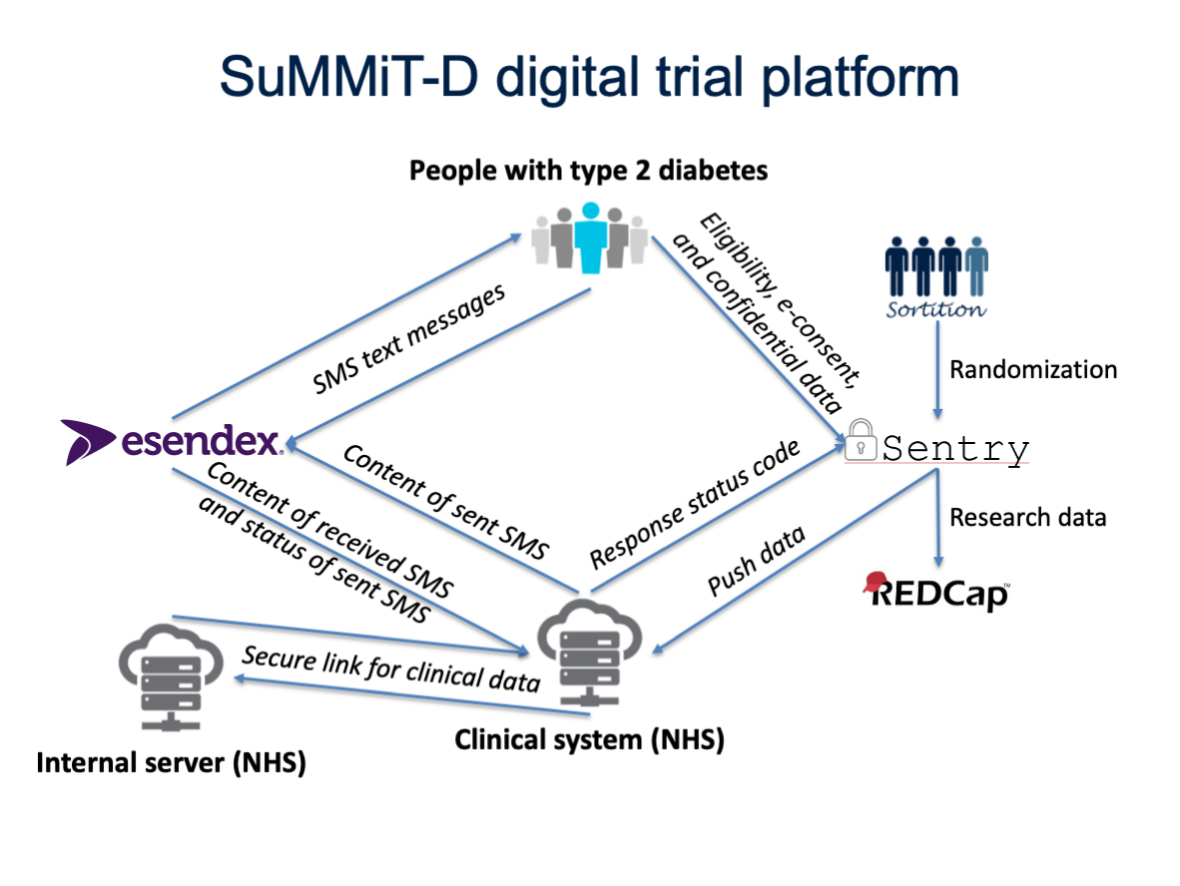
Overview of systems developed for the Support through Mobile Messaging and Digital Health Technology for Diabetes study. Sentry: Secure entry; SuMMiT-D: Support Through Mobile Messaging and Digital Health Technology for Diabetes.

Figure 1 shows the system architecture designed for the SuMMiT-D study. The design work required the coordination of the following platforms and resources: Sentry, an online-based recruitment system developed and managed by the Primary Care Clinical Trials Unit, University of Oxford; the mobile phones used by the participants to send and receive SMS messages; a web interface accessible by the system users (ie, system administrators and research staff) to manage the clinical system; and Esendex [25], an SMS engine provider that enabled delivery and receipt of text messages to and from participants. The system was also designed to integrate alerts and personalization of messages based on primary care electronic health record (EHR) data. Platforms and protocols were required to meet the standards of data and security management [26]. Operating procedures were designed to achieve secure and independent management of the system as well as the storage of data.

#### Clinical System

The back end (ie, the data access layer that provides access to data stored in a database) of the clinical system was developed using the CakePHP web framework [27], and the associated database tables were developed using the relational database management system MySQL [28], whereas the front end (ie, the presentation layer that formats and delivers information for further processing or display) was developed using three core technologies of the WWW [29]: HTML [30], Cascading Style Sheets [31], and Javascript [32]. These open-source technologies were chosen because of ease of use, fast iteration, and adherence to communication standards. The core component of the clinical system was implemented using CakePHP. The CakePHP web framework [27] has several advantages: it is an open-source platform that supports the PHP programming language [33]; it requires almost no preconfigurations, as most of the settings and options are auto-detected; it implements a model-view-controller pattern, which divides the developing user interfaces into 3 interconnected elements: model, view, and controller; it offers a wide range of built-in plug-ins (ie, software components that add specific features to an existing computer program) to enable customization; and it supports fast connectivity with database systems. All these characteristics make it particularly suitable for the reliable and extendible implementation of web-based services.

To meet both the trial and the NHS security and privacy requirements and to allow clinical implementation once the whole trial ends, the clinical system has been deployed in a server managed by the Oxford University Hospitals NHS Foundation Trust. The server is located behind the firewall managed by the local information management and technology team at the Oxford University Hospitals NHS Foundation Trust and hosted within the NHS Health and Social Care Network [34]. Access to this system is password protected and available only to registered users. All data (ie, database tables and participants’ GP records) are stored separately on another internal server, which is inaccessible directly from the internet and communicates with the main front-end server via a password-protected, Transport Layer Security (TLS) [35] encrypted channel.

**Figure 2:**
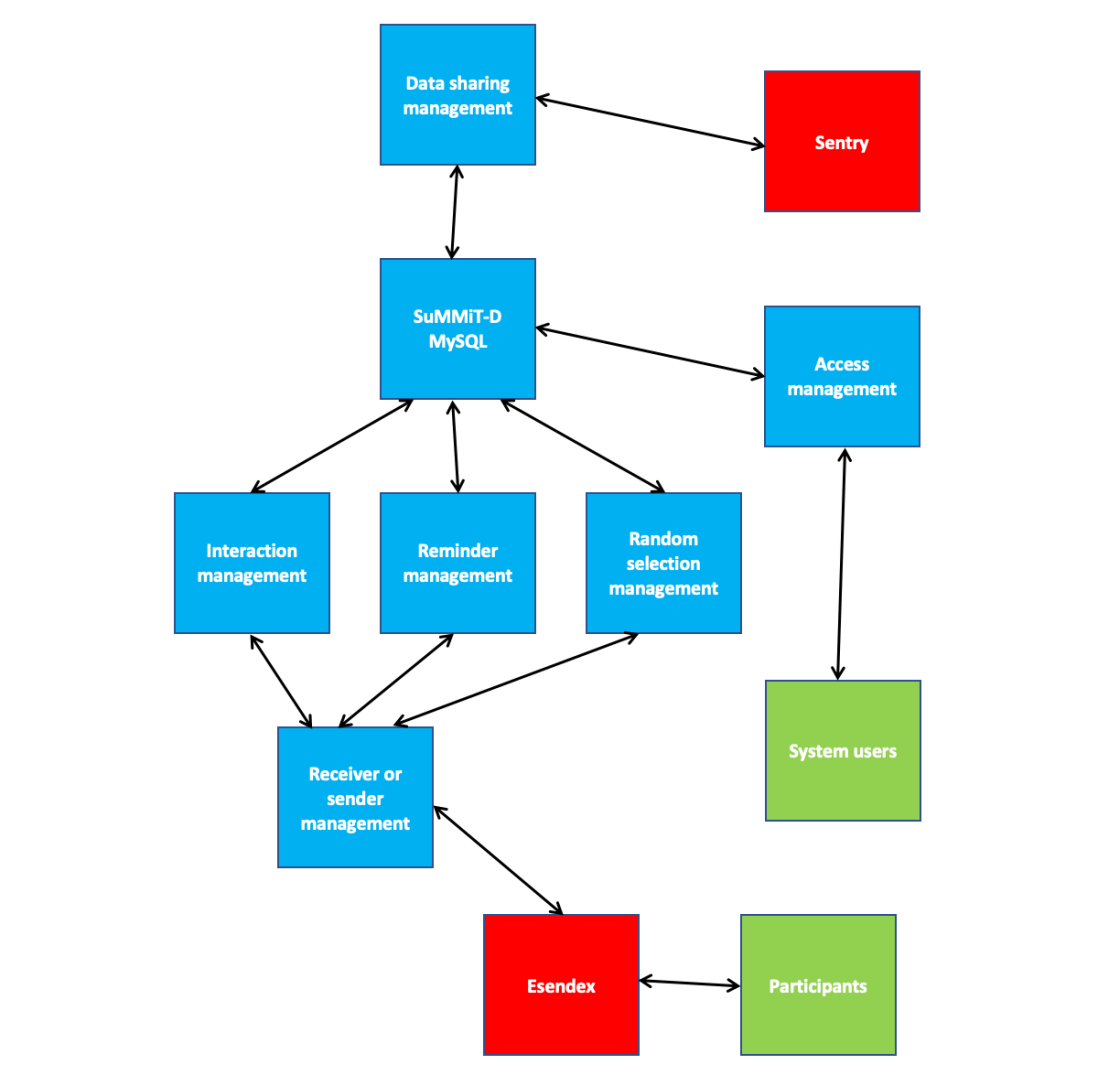
Support through Mobile Messaging and Digital Health Technology for Diabetes architecture. Sentry: Secure entry; SuMMiT-D: Support Through Mobile Messaging and Digital Health Technology for Diabetes.

Figure 2 shows the architecture of the clinical system with various key components that have been developed. The various components of the system include the following: *Access Management*, *Data Sharing Management*, *Receiver and Sender Management*, *Random Message Selection Management*, *Reminder Management*, *Interaction Management*.

Participants can interact with the SuMMiT-D system by sending SMS messages from their mobile phones to a virtual mobile number (provided by Esendex) using one of the predefined commands. Esendex provides two ways of interacting with their SMS engines: (1) using the toll-free number *0800* or (2) using a virtual mobile number. For using the toll-free number *0800*, each command needs to start with a specified keyword (eg, *DIA*) for Esendex to identify the system with which the user wants to interact. In using a virtual mobile number, a virtual number is exclusively associated with one system, so that the routing keyword is not required. In this instance, the user bears the cost of sending SMS messages. Although the latter method represents a cost to the user, it has the following advantages: the cost of sending a text message is acceptable to participants; text messages from participants will always reach the SuMMiT-D system; and if the former method is used and the specified keyword has been forgotten or mistyped, the delivery of messages to the SuMMiT-D system will fail. System users (ie, health care professionals, research staff, and system administrators) engage with the clinical system using an internet-enabled computing device (eg, a smartphone, a tablet, or a computer).

#### Interaction Management

The *Interaction Management* module determines how the implemented algorithms in the SuMMiT-D system tailor messages according to each individual’s preferences. Each time a participant sends a text via Esendex, the system (1) retrieves the original text from Esendex via the receiver or sender management; (2) parses and interprets the text referring to preferences; and (3) generates a response to be pushed back, for Esendex to finally send it to the corresponding participant.

The study participants can send predefined commands to the SuMMiT-D system. A command in the context of text messages refers to one or several words corresponding to one or more actions on the SuMMiT-D receiver end. In the SuMMiT-D system, the commands correspond to a list of settings that participants can change according to their preferences, for example, when to receive messages in a day and like or dislike the last received message to express interest in more or fewer messages of the same kind in the future. Other possible interactions include initial registration with the system, pausing messages for X weeks, restarting from being paused, completely stopping receiving messages, reviewing, and rating received messages, and requiring help instructions on how to interact with the system. The full list of commands is listed in Table 1.

**Table 1:** List of commands available in the Support through Mobile Messaging and Digital Health Technology for Diabetes system and the corresponding descriptions.

Study and commands		Description
**Common to both studies**		
	Register *names*	Register with the SuMMiT-D system
	Start	Start receiving messages again
	Stop	Stop receiving messages completely (Need to contact the SuMMiT-D study team to restart the messages)
	AM	Receive messages in AM (from 9 AM to 12 PM)
	PM	Receive messages in PM (from 12 PM to 6 PM)
	Help, Help 1, Help 2	Help messages for using the list of commands
**Pilot**		
	Less or More	Receive fewer or more messages similar to the last received one
	Like or Dislike	Like or Dislike the last received message
	Easy or Hard	The last received message is easy or hard to understand
	Useful or Not Useful	The last received message is useful or not useful
	Help 3	Help messages for using the list of commands
**Feasibility**		
	Like or Dislike	Receive fewer or more messages similar to the last received one

#### Reminder Management

Besides instantaneous interactions, a participant also received scheduled text messages during the study. The *reminder management* module schedules text reminders. During the pilot study, to encourage the participants to review and rate the randomly selected messages sent to them, a prompt text message was sent to each participant every week (ie, a reminder to use the commands *Like or Dislike, Easy or Hard*, and *Useful or Not Useful*). For the pilot study, after 4 weeks of participation, a reminder was sent on how to use the commands *More or Less*; whereas for the feasibility study, 4 weeks after randomization, a reminder was sent on how to use the commands *Like or Dislike*. In addition, at the beginning and in the middle of both studies, a participant would receive a text reminder on how to use the *Help* commands. Other types of engagement messages were a happy birthday text message and a reminder on the day of world diabetes day celebration (14th November).

#### Random Selection Management

The SuMMiT-D study participants received a number of brief messages sent periodically. Two types of brief SMS messages were used: one type is based on behavior change techniques (BCTs), and the other type is based on general lifestyle advice [36-38].

During the pilot study, in which all participants belonged to one group, these messages were sent three times a week. For every five continuous BCT-based messages, one general lifestyle message was sent.

During the feasibility study, participants were randomized to either the control or intervention groups. For those in the control group, only control messages were sent at a frequency of approximately once a month. The control messages here refer to general messages that are irrelevant to medicine management. For those in the intervention group, two types of messages (BCT and general lifestyle) were sent at a frequency of 3 messages per week (1 general lifestyle message, for every 2 continuous BCT-based messages). The increased frequency of lifestyle messages was based on the feedback of the participants in the pilot study. Examples of SMS text messages used in the SuMMiT-D feasibility study are shown in Table 2.

**Table 2:** Examples of SMS text messages used in the Support through Mobile Messaging and Digital Health Technology for Diabetes feasibility study.

Message category		Example messages
**Behavior change technique**		
	1.4 Action Planning	Plan when, where and how you are going to take your medication
	2.3 Selfmonitoring	Find a way to split your tablets into days so you notice when you have forgotten to take your tablets.
	7.1 Prompts or cues	It can be difficult to remember to take your tablets. Why not set an alarm to remind you to take them?
	G Health care system related concerns	Lots of questions? Check who the best person to see might be.
**General lifestyle**		
	Signposting	Want to hear what other people with diabetes think? You can hear different people discuss their experiences at healthtalk online.

For the pilot study, there were a total of 157 unique BCT messages used, and they were from 30 different BCT groups, whereas there were 35 unique general lifestyle messages. On the basis of the feedback (ie, the message ratings) of the participants in the pilot study, the messages used in the feasibility study have been edited accordingly: there were a total of 170 unique BCT messages used, and they were from 30 different BCT groups, whereas there were 35 unique general lifestyle messages and 6 unique control messages. More detailed information on the messages used in the pilot and feasibility studies can be found in the studies by Farmer et al [24] and Bartlett et al [39].

The algorithm below describes the methodology for sending these SMS messages:

Suppose there are t different groups of BCTs as: {*B*_1_, *B*_2_, ..., *B*_*t*_}, with the *i*-th (1≤*i*≤*t*) BCT group having *n_i_* different SMS texts, that is, 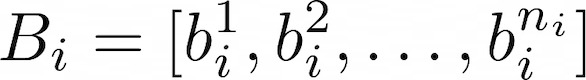. This results in *n* unique BCT SMS texts, where 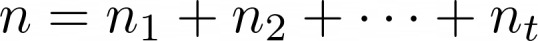. There are also *m* different general lifestyle SMS texts 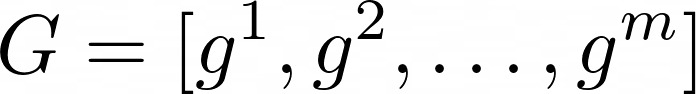. As a result, there are *n+m* unique SMS messages in the system, which can be represented as {*B*_1_, *B*_2_, ..., *B*_*t*_, *G*}. The SMS messages sent to each participant follow this rule: for each week (ie, 7 days), 3 messages are sent, and they are sent with an even time gap; in addition, after continuously sending *q* BCT-based SMS messages, 1 general lifestyle-based message should be sent (*q*=5 for the pilot study and *q*=2 for the feasibility study according to the participants’ feedback from the pilot study).

The SMS messages sent to each participant were randomly selected as follows: for each participant, all *t* different BCT groups of messages have the same probability. To ensure that a different message was received each time, the system excluded all the previously sent messages and the most recently used BCT group and selected a message from the remaining messages and BCT groups. The same approach was used when sending general lifestyle-based SMS messages. The above process is enabled only for those participants who were scheduled to receive a message and only within the time slot selected by each participant.

In addition to the above process, the SuMMiT-D system tailors and personalizes the messages for each participant. Tailoring and personalization were performed according to the text responses from each participant. In the first instance, upon receiving *Pause X*, *Start*, and *STOP* from a participant, messages could be paused for X weeks, restarted, or completely stopped, respectively.

Upon receiving *More* and *Like* from a participant in the pilot and feasibility studies, respectively, after she or he receives a BCT-based message (ie, 
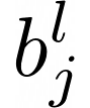
, where 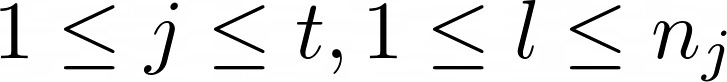), another message from the same BCT group (ie, 
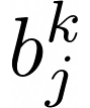
, where 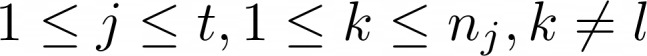) is scheduled to be sent to this participant after 2 weeks. In addition, all the messages in the same BCT group (ie, 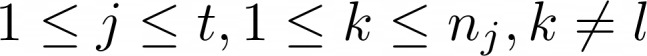) would have doubled the chance (ie, increase 100% probability) to be selected and sent to this participant in the future. Upon receiving *Dislike* from a participant in the feasibility study, after she or he receives a BCT-based message (ie, 
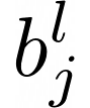
, where 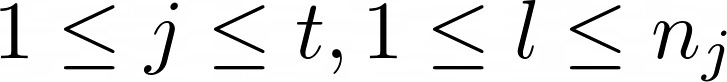,), all the messages in the same BCT group (ie, 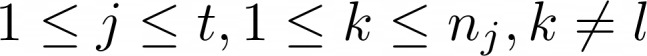) would have halved the chance (ie, decrease 50% probability) to be selected and sent to this participant in the future. For details, please refer to the pseudocode listed in Multimedia Appendix 1.

Upon receiving *AM* and *PM* from a participant, the message delivery time slot to this participant was changed accordingly.

#### Receiver or Sender Management

The purpose of this component is to send and receive messages to and from participants. This management component employs a third-party mobile messaging system (Esendex [25]) to act as a bridge between the participants and the clinical system. To dispatch SMS messages to the participants’ mobile phones, they are sent to the Esendex system’s application program interface (API) by the clinical system. The information exchanged between the 2 systems includes the target mobile number, the message context to be sent, a unique message ID, and a key code to authorize this message to be sent. This communication is established over an encrypted HTTP channel over TLS [35]. After 5 hours, the clinical system again connects to Esendex’s API and then checks the delivery status of the message using the unique message ID, which is returned by Esendex during the first connection. This process allows tracking of the delivery status of every sent message. Each message delivery status (ie, *Delivered, Sent, Failed, Expired, Cancelled,* and *Partially Delivered*) is stored by the clinical system, along with the corresponding message information.

For messages whose delivery fails (ie, a delivery status of *Failed*, *Expired*, *Cancelled*, and *Partially Delivered*), another attempt is scheduled immediately. After further 2 failed redelivery attempts, an automatic email is sent to the system users, whose role is to be either administrators or researchers, to notify this failed delivery and suggest the possibility that the participant may have changed this mobile number.

The clinical system checks Esendex’s API at an interval of every 30 seconds. This very short period was selected to provide a sense of timeliness of the response to (potential) participants and to maintain a high engagement level.

#### Data Sharing Management

The eligible candidates were screened and then invited to the SuMMiT-D study (full details of the recruitment process have been reported elsewhere [24]). To participate in any SuMMiT-D study, patients had to express their interest with a text message (ie, Register *names*) to one of the virtual numbers provided by Esendex. Once the text was received, an automatic reply was sent back. This handshake process guaranteed that the mobile number was valid. For the pilot study, the recruitment process was completed over the phone, the paper consent form was sent and returned via post, and personal information was directly entered into the SuMMiT-D system via a web interface. For the feasibility study, to add another level of security and automation, the recruitment process was performed electronically via a system called Sentry (Secure entry) [40]. The consent form could then be completed electronically. Once the recruitment process for a participant was completed, all personal details were securely stored in REDCap [41]. To enable data sharing of personal details required to be shared with the clinical system, data were pushed from the Sentry system to the clinical system via HTTPs [42]. During this data transaction, the mobile number that the participant used to express interest was used as the matching key. The following personal details were shared by Sentry and stored in the clinical system: *first name, last name, mobile number, GP practice name, date of birth, preferred name, gender, NHS number, whether using a smartphone*, and *participant study ID*. As Sentry also implemented the randomization algorithm for determining the control or intervention group, it shared also the randomization result so that the SuMMiT-D system would know whether a participant data that had been pushed from Sentry to the clinical system belonged to the control or intervention group.

#### Access Management

System users can log in to the SuMMiT-D system using their personal usernames and passwords. Users are categorized according to 3 levels of privileges that determine their access rights to the information and functions available. The system administrators (*Admin*) get access to the entire system and all its functionalities; the study researchers (*Researcher*) get full access to the information of participants and GP practices; and the research nurses, trail manager, and coordinator (*User*) get only a read-only view of the information of participants and GP practices.

The web pages that a system user can access depend on the privilege level, which is determined by the log-in credentials. Table 3 provides the main views that a user can access according to their privilege level and a brief description for each of those views.

**Table 3:** Privilege levels and the corresponding functionality available at each of these privilege levels.

Functionality	Privilege level		
	Admin	Researcher	User
Add system users (admin, researcher, user) List of system users Detailed view or edit of system users	✓^a^	—^b^	—
Add participants Add GP^c^ practices Detailed edit of participants Detailed edit of GP practices List of sent or received SMS Detailed view of sent or received SMS	✓	✓	—
List of participants Search participants List of GP practices Detailed view of participants Detailed view of GP practices Detailed view or edit of own details	✓	✓	✓

^a^Function is available at this level.

^b^Function not available at this level.

^c^GP: general practitioner.

### Development Approach

The SuMMiT-D system was developed in collaboration with health care professionals, the study team, the software development team, and a group of patient representatives. Both test GP practices and patients were set up during the early development process, so that the system could be evaluated for its reliability and to allow software bugs to be identified and fixed.

### Defined Metrics

To evaluate the performance of the SuMMiT-D system, the following 3 metrics were defined: *response rate, keyword percentage, and rate versus prompt*.

#### Response Rate

To determine whether a participant was active in the study, the response rate of SMS messages for each participant was calculated according to:


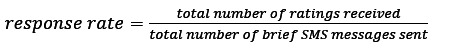

(**1**)

where, for the pilot study,


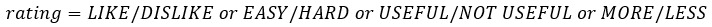

(**2**)

whereas, for the feasibility study,


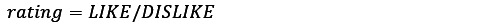
(**3**)

#### Keyword Percentage

In the pilot study, to evaluate the usability of a keyword-based system, a fictitious keyword was selected. Participants were asked to submit all commands after using the DIA keyword. The keyword (DIA) percentage is calculated according to:


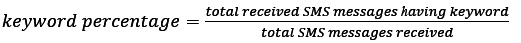

(**4**)

#### Rate Versus Prompt

In the pilot study, to review whether the prompt messages could improve the total number of responses (ie, rating) received, the ratio of number of response messages to the number of prompt messages is calculated as:


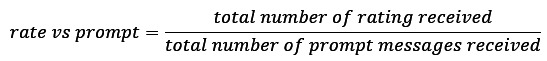

(**5**)

Where rating is calculated according to equation 2.

## Results

### Pilot Study

For the pilot study, 48 participants were recruited, with a median age of 64 years (first quartile and third quartile [*Q*_1_, *Q*_3_: 54.5, 69]). Among the 48 participants, 19 (40%) were from the Greater Manchester region and 29 (60%) were from the Thames Valley region.

#### Duration of Study and Admission Process

The first participant expressed interest on April 24, 2018, and the last participant expressed interest on August 24, 2018. The first participant started to use the SuMMiT-D system on May 8, 2018, whereas the last participant started to use the system on September 17, 2018. The first participant stopped using the SuMMiT-D system (ie, either finished or withdrew from the study) on August 11, 2018, whereas the last one stopped on January 31, 2019.

An admission process duration refers to how long (in days) it took the participants from expressing interest (ie, sending the *Register* command to register with the system) to fully start the SuMMiT-D study (ie, to begin receiving regularly sent messages). The admission process duration here has a median value of 21.07 days (*Q*_1_*, Q*_3_: 17.31, 24.93).

Two participants withdrew from the study at days 23 and 67 (withdrawal rate of 4%). At 3 months (13 weeks) after using the system, 19% (9/48) participants chose to stop, whereas the remaining 77% (37/48) decided to continue.

In addition, a total of 11 *STOP* commands have been received, which are in line with the results above: 2 participants withdrew and 9 stopped at 3 months after using the system. Only 2 Pause *X* commands were received: one chose to pause the messages for 2 weeks and the other paused for 4 weeks.

#### SMS Message Response Rate of Participants

The metric here is defined as the *response rate*, which is calculated according to equations 1 and 2. The median *response rate* was 71% (*Q*_1_*, Q*_3_: 53%, 111%). This actual response rate is lower than what might have been expected, as there was a weekly reminder (ie, the prompt message encouraging participants to rate received messages).

#### Participants Including the Keyword DIA in Their Responses

Both toll-free and virtual numbers are available for SMS-based studies to interact with participants. The first approach generally shares the same toll-free number across different systems and, thus, requires the use of a keyword to identify the SMS texts received from users. However, the additional keyword may represent a challenge for some participants, as they may forget to add the keyword at the start of a message, and as a result, such a message will be missed at the corresponding system end completely.

The metric here is defined as the *keyword percentage*, which is calculated according to equation 4. The median *keyword percentage* was 93% (*Q*_1_*, Q*_3_: 87%, 98%). The histogram for the keyword percentage is shown in Multimedia Appendix 2. Although the keyword (*DIA*) was used quite frequently, some messages would have still not been received on the SuMMiT-D system side.

#### Prompt Messages Versus Message Responses

A weekly reminder was sent to the participants in the pilot study in the form of a prompt message asking to rate the received messages. To review whether this could improve the total number of responses (ie, rating) received, the metric *rate versus prompt* is calculated as described above equation 5. The r*ate versus prompt* has a median value of 280% (*Q*_1_*, Q*_3_: 215%, 434%). The rate versus prompt is also lower than expected, which is in line with the results shown above.

**Figure 3:**
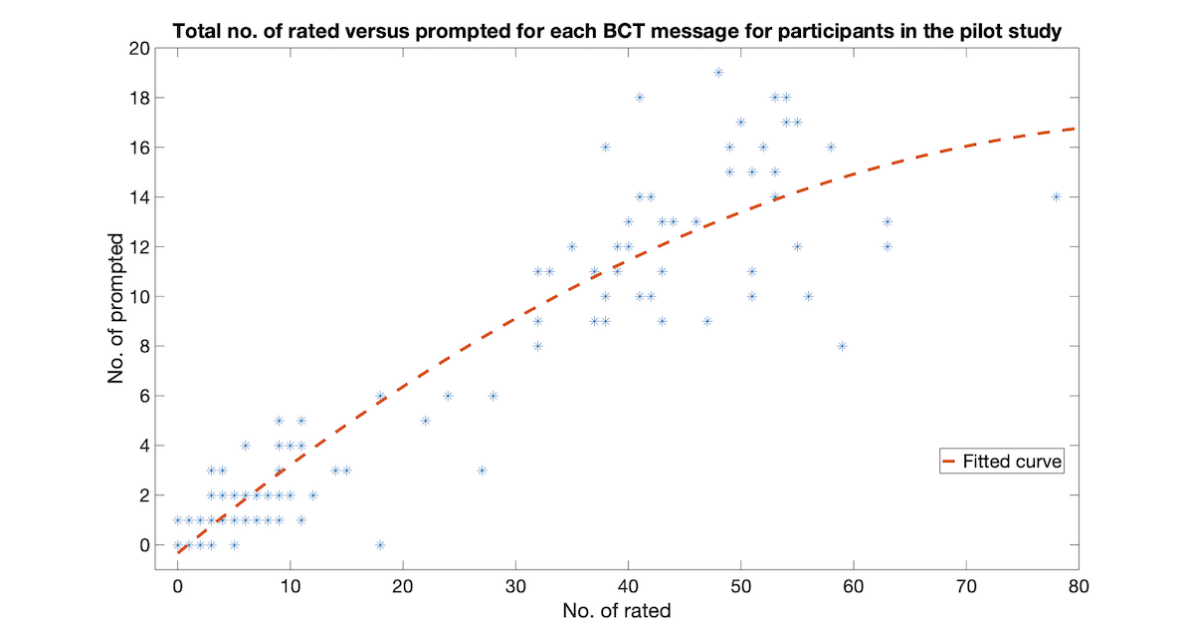
The total number of rated versus prompted for each behavioral change therapy message for participants in the pilot study.

In addition, for each BCT message, the total number rated against the total number prompted is summarized and shown in Figure 3, with a quadratic polynomial curve fitted to the data points. From the fitted curve, prompt messages can potentially improve the total number of responses (ie, rating) received.

#### Comparison Among the Preference Groups of Messages Responses

**Figure 4:**
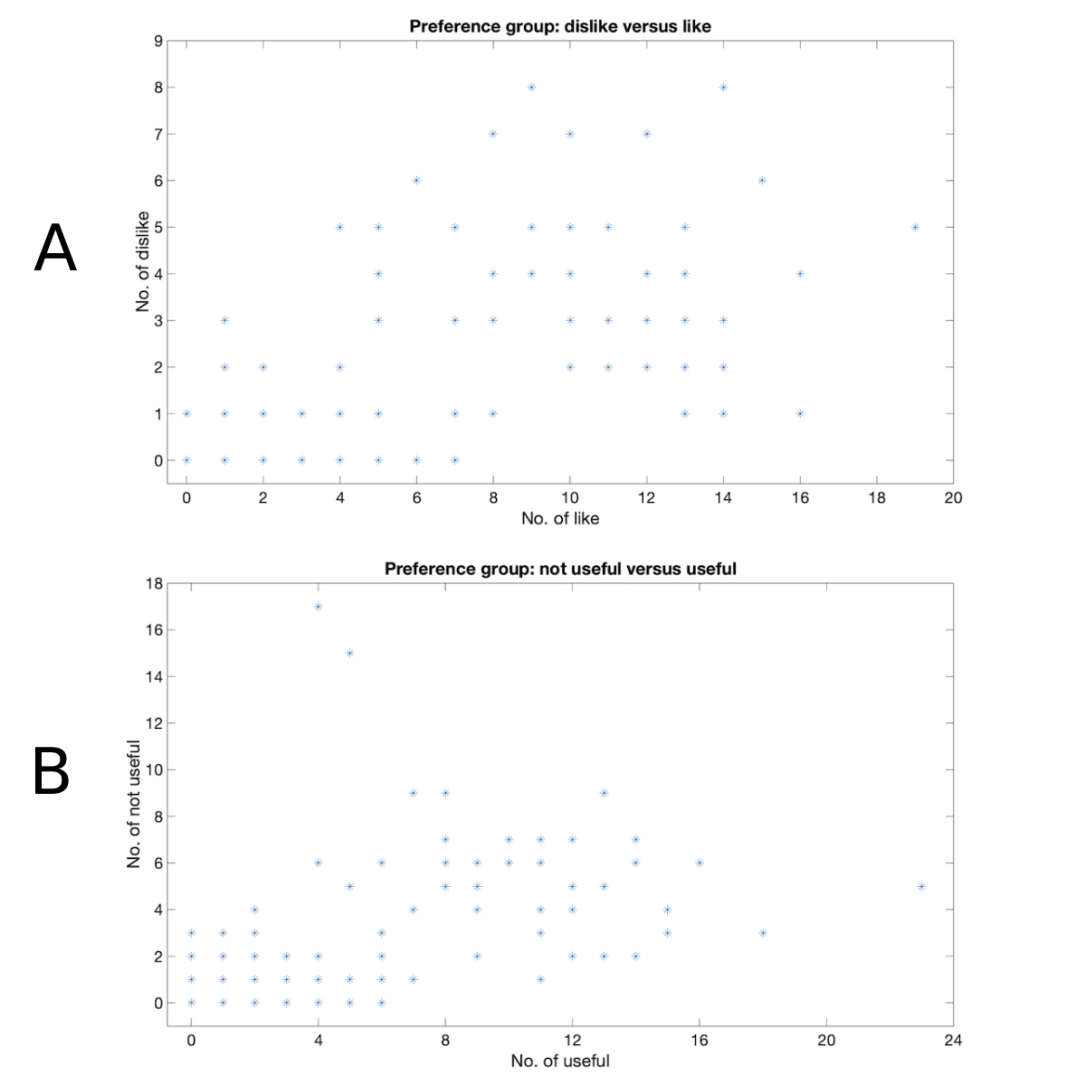
Different preference groups among the SMS message response rating for participants in the pilot study. A: Preference group: Dislike vs Like; B: Preference group: Not useful vs Useful.

To compare which preference groups (ie, Like or Dislike, Easy or Hard, Useful or Not Useful, and More or Less) are mostly used by participants in the pilot study, the total numbers of Like or Dislike, Easy or Hard, Useful or Not Useful, and Less or More were counted. The results are shown in Figure 4. From the results, it can be concluded that the vast majority of the BCT messages sent in the pilot study received a positive rating (ie, Like and Useful).

### Feasibility Study

For the feasibility study, a total of 209 participants (with a median age of 65 years, *Q*_1_*, Q*_3_: 56, 71; with 86/209, 41.2% female) were recruited and randomized. Of these, 106 were randomized into the control group, whereas the remaining 103 were randomized into the intervention group. Of the 209 participants, 52 (24.8%) were from the Greater Manchester area and 157 (75.1%) were from the Thames Valley area.

#### Duration of Study and Admission Process

The first participant expressed interest on December 3, 2018, and the last one expressed interest on March 20, 2019. The first participant started to use the SuMMiT-D system on December 10, 2018, whereas the last participant started to use the system on April 16, 2019. The first participant stopped using the system (ie, either finished or withdrew from the study) on March 4, 2019, whereas the last one stopped using the system on October 16, 2019.

The admission process duration here has a median value of 8.76 days (*Q*_1_*, Q*_3_: 3.94, 15.07).

A total of 11 participants withdrew from the study (10 from the intervention group): 8 of them stopped the messages via sending the *STOP* commands, whereas the remaining 3 called the study team to ask for withdrawal. A total of 7 *Pause X* commands were received, and they were from 4 different participants.

#### SMS Message Response Rate of Participants

The metric here is the *response rate*, which is calculated according to equations 1 and 3. The median *response rate* was 17% (*Q*_1_*, Q*_3_: 13%, 22%). It can be concluded that there is a much lower response rate for the participants in the feasibility study, as there was no weekly prompt message sent in the latter study.

#### Like Versus Dislike Among Message Responses

**Figure 5:**
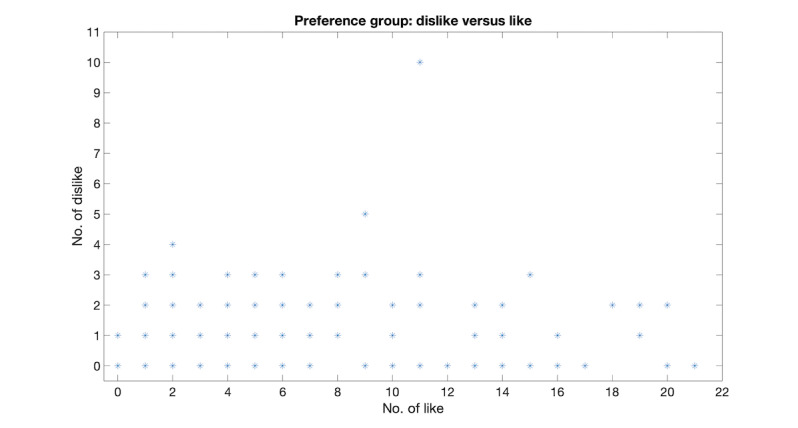
Like versus Dislike among the SMS message response ratings for participants in the feasibility study.

To compare which BCT message is mostly liked or disliked by participants in the feasibility study, the total number of Like or Dislike was counted for all the BCT messages, and the results are shown in Figure 5. In the same way as Figure 4, Figure 5 shows that the great majority of the BCT messages sent in the feasibility study receive a positive rating (ie, Like).

#### Impact of Like or Dislike Reminder

**Figure 6:**
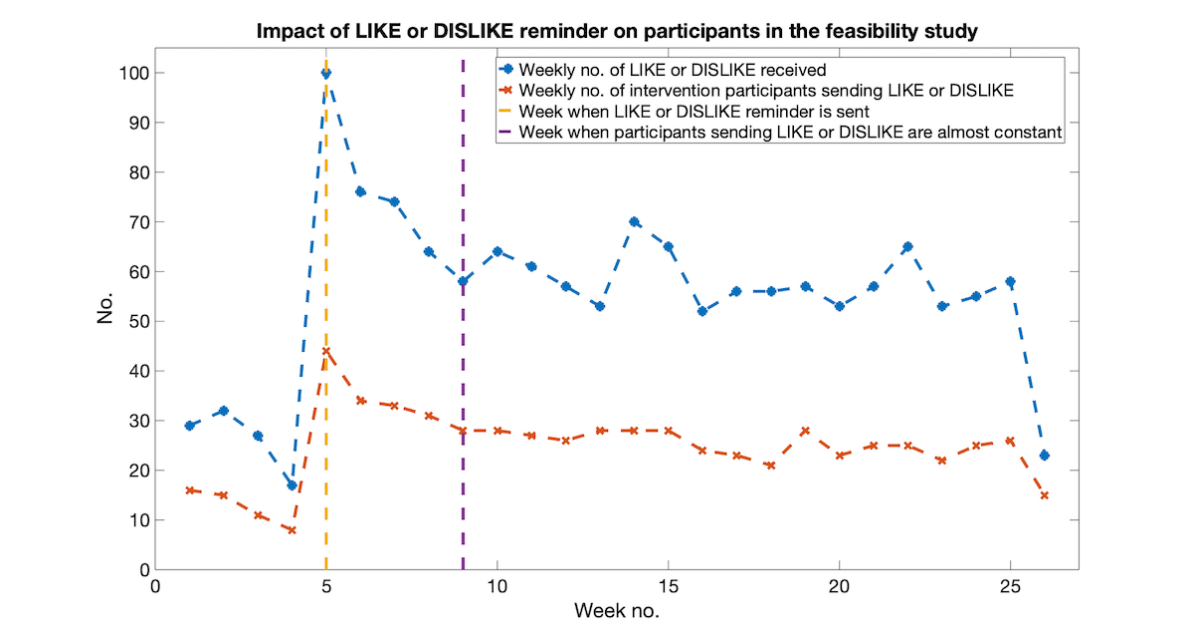
Impact of Like/Dislike reminder on participants in the feasibility study.

Unlike the pilot study, there were no weekly prompt SMS messages (ie, encouraging participants to rate all the received SMS messages) sent in the feasibility study. To remind the participants in the intervention group to use the Like or Dislike commands, a reminder of how to use these commands was sent to them when they had been in the study for 4 weeks. To determine the impact of Like or Dislike reminders (ie, how long the reminders will have an effect on the participants), the following 2 numbers have been plotted: the total number of Like or Dislike commands received per week and the number of participants sending Like or Dislike commands per week. The results are shown in Figure 6. From the results, it can be seen that in week 5 (ie, just after the reminders were sent), these 2 numbers reached their peak values. From week 9 (ie, 4 weeks after the reminders were sent), the number of participants sending Like or Dislike commands back per week remained almost constant. This suggests that the impact of Like or Dislike reminders persists for approximately 4 weeks.

## Discussion

In these pilot and feasibility studies for the SuMMiT-D brief messaging, mobile phone–based intervention, participant withdrawal rates were 4.2% and 5.3%, respectively. In addition, from the message responses, it was concluded that most of the participants gave positive feedback to the BCT messages they received (ie, useful and like). Furthermore, when offered the opportunity to continue for another 3 months in the pilot study, 37 out of 46 participants chose to stay. These promising results suggest that the SuMMiT-D system is robust, user-friendly, useful, and positive for most participants.

Comparing the length of the admission process (registration and sign up), it can be seen that there is a significant improvement in the feasibility study compared with the pilot. This may be explained by changing the recruitment method from regular mail (ie, manually sending and receiving all forms: registration, questionnaires, and consent) to online recruitment.

One common issue in both studies is that the response rate of participants to the messages sent to them was lower than expected. This might have been associated with the demographics of those recruited to the studies (with a median age of about 65 years) where participants might not have been familiar with the use of mobile phones. In the pilot study, a weekly prompt message was sent to participants to encourage them to rate received messages. In the feasibility study, a one-time Like or Dislike reminder describing how to use the Like and Dislike commands was sent to participants. Our findings suggest that (1) prompt messages can potentially improve the total number of responses received and (2) a reminder encouraging participants to Like or Dislike messages has an effect persisting for about 4 weeks before a participant returns to their normal response rate. Potentially higher response rates may be obtained from participants if they were sent the reminder every 4 weeks.

The SuMMiT-D system uses brief messages as interventions to provide education and support self-management for people with type 2 diabetes. The brief messages are delivered through mobile phone–based SMS text messages. These patients are often concerned about starting new diabetes medicines or face difficulties in taking their medicines regularly. To enhance the support for patients’ self-management, these brief messages were personalized and tailored for each participant individually, according to each patient’s own preference and their feedback to previously received SMS text messages.

The SuMMiT-D system offers a model for technology-based self-management support [30,31], as it shows the clinical efficacy and cost-effectiveness of a text messaging intervention, compared with the usual care, for people with type 2 diabetes. In addition, the SuMMiT-D system is not only an effective tool for type 2 diabetes, but it could also be extended to other long-term conditions (eg, hypertension).

All the information and feedback that we gathered in each of the studies was carried out iteratively to improve the functionalities of the system. From the pilot study, we learned the following: (1) a recruitment process via regular post was time consuming and imposed a high workload from the study team, and in the feasibility study, we introduced the Sentry system to allow a digital (online) recruitment process, and (2) according to the participants’ feedback, they would have liked to receive the general lifestyle messages more frequently, and we increased the ratio of the general lifestyle and BCT messages from 1 to 5 in the pilot study to 1 to 2 in the feasibility studies.

In the SuMMiT-D pilot and feasibility studies, we have demonstrated the successful delivery of interventions to more than 200 participants via SMS text messages. In the main SuMMiT-D study, to further enhance the support for patients’ self-management, their EHR data will be routinely shared and collected via a GP data provider. EHR data, such as medication possession ratio and glycated hemoglobin, will be used to tailor the intervention messages. In addition, more automation in the recruitment process will be introduced, such that the overall workload is reduced.

## Appendix

Multimedia Appendix 1 Algorithm 1 pseudocode for receiving “More”/“Like” or “Dislike” after sending a behavior change technique message to a participant.

Multimedia Appendix 2 Histogram plot of the keyword percentage keyword percentage among the SMS SMS message responses for participants in the pilot study.

## Abbreviations

API: application program interface
BCT: behavior change technique
EHR: electronic health record
GP: general practitioner
NHS: National Health Service
NIHR: National Institute for Health Research
*Q*_1_, *Q*_3_: first quartile and third quartile
SuMMiT-D: Support through Mobile Messaging and Digital Health Technology for Diabetes
TLS: transport layer security
