# Correction: System Architecture for "Support Through Mobile Messaging and Digital Health Technology for Diabetes" (SuMMiT-D): Design and Performance in Pilot and Randomized Controlled Feasibility Studies

**DOI:** 10.2196/29451

**Published:** 2021-04-09

**Authors:** Yuan Chi, Carmelo Velardo, Julie Allen, Stephanie Robinson, Evgenia Riga, David Judge, Lionel Tarassenko, Andrew J Farmer

**Affiliations:** 1 Institute of Biomedical Engineering Department of Engineering Science University of Oxford Oxford United Kingdom; 2 Nuffield Department of Primary Care Health Sciences University of Oxford Oxford United Kingdom

In “System Architecture for “Support Through Mobile Messaging and Digital Health Technology for Diabetes” (SuMMiT-D): Design and Performance in Pilot and Randomized Controlled Feasibility Studies” (JMIR Form Res 2021;5(3):e18460) two errors were noted after publication.

In the originally published version, under the section “Random Selection Management,” an inline graphic was not inserted in the text in the following sentence:

In addition, all the messages in the same BCT group (ie, Inline graphic 10) would have doubled the chance (ie, increase 100% probability) to be selected and sent to this participant in the future.

The graphic has been added to this sentence in the corrected version.

Two extraneous inline graphics were also inadvertently published in the Results section due to a system error. These have been removed from the corrected version.

The correction will appear in the online version of the paper on the JMIR Publications website on April 9, 2021, together with the publication of this correction notice. Because this was made after submission to PubMed, PubMed Central, and other full-text repositories, the corrected article has also been resubmitted to those repositories.

