# Supplementary material for: System Architecture for "Support Through Mobile Messaging and Digital Health Technology for Diabetes" (SuMMiT-D): Design and Performance in Pilot and Randomized Controlled Feasibility Studies
Source: JMIR Form Res. 2021 Mar 26;5(3):e18460. doi: 10.2196/18460 (PMC8034865; doi:10.2196/18460)
Supplement: Multimedia Appendix 1 [file formative_v5i3e18460_app1.docx]

### Appendix 1

| **Algorithm 1** Pseudocode for Receiving “MORE”/“LIKE” or “DISLIKE” after Sending a BCT Message to a Participant |
| --- |
| **Require:** receive “MORE”/“LIKE” or “DISLIKE” after sending a BCT message $b_{j}^{l}$ ($1\leq j\leq t$, $1\leq l\leq nj$) to a participant  **if** receive “MORE”/“LIKE” after sending the BCT message $b_{j}^{l}$ **then**  **if** the BCT group $B_{j}= [b_{j}^{1},b_{j}^{2},\cdot\cdot\cdot, b_{j}^{n_{j}}]$ is in the “MORE”/“LIKE” list for this participant **then**  no further action  **else if** the BCT group $B_{j}= [b_{j}^{1},b_{j}^{2},\cdot\cdot\cdot, b_{j}^{n_{j}}]$ is in the “DISLIKE” list for this participant **then**  remove the BCT group $B_{j}$ from the “DISLIKE” list for this participant  increase100%probability of the BCT group $B_{j}$ to be selected and sent to this participant  **else**  add the BCT group $B_{j}$ to the “MORE”/“LIKE” list for this participant  increase100%probability of the BCT group $B_{j}$ to be selected and sent to this participant  **end if**  **else if** receive “DISLIKE” after sending the BCT message $b_{j}^{l}$ **then**  **if** the BCT group $B_{j}= [b_{j}^{1},b_{j}^{2},\cdot\cdot\cdot, b_{j}^{n_{j}}]$ is in the “MORE”/“LIKE” list for this participant **then**  remove the BCT group $B_{j}$ from the “MORE”/“LIKE” list for this participant  decrease 50% probability of the BCT group $B_{j}$ to be selected and sent to this participant  **else if** the BCT group $B_{j}= [b_{j}^{1},b_{j}^{2},\cdot\cdot\cdot, b_{j}^{n_{j}}]$ is in the “DISLIKE” list for this participant **then**  no further action  **else**  add the BCT group $B_{j}$ to the “DISLIKE” list for this participant  decrease 50% probability of the BCT group $B_{j}$ to be selected and sent to this participant  **end if**  **end if** |
