# Supplementary figures and images for: System Architecture for "Support Through Mobile Messaging and Digital Health Technology for Diabetes" (SuMMiT-D): Design and Performance in Pilot and Randomized Controlled Feasibility Studies
Source: JMIR Form Res. 2021 Mar 26;5(3):e18460. doi: 10.2196/18460 (PMC8034865; doi:10.2196/18460)

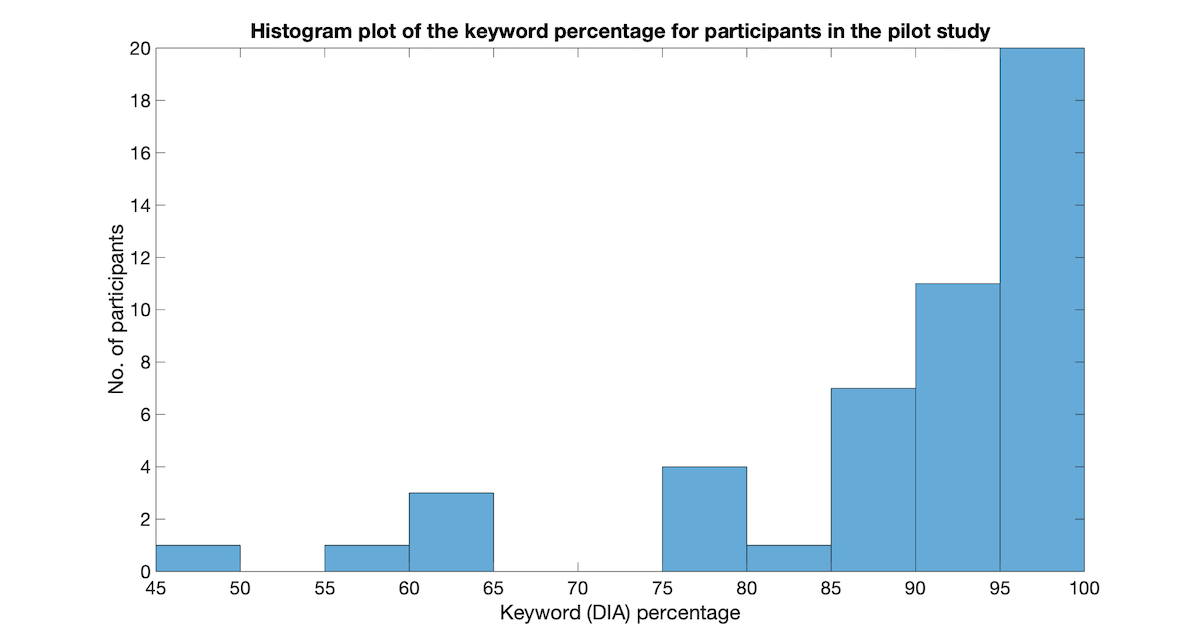

Supplement: Multimedia Appendix 2 [file formative_v5i3e18460_app2.png]
